# Ferroptosis: A potential target for the intervention of intervertebral disc degeneration

**DOI:** 10.3389/fendo.2022.1042060

**Published:** 2022-10-20

**Authors:** Lu-Ping Zhou, Ren-Jie Zhang, Chong-Yu Jia, Liang Kang, Zhi-Gang Zhang, Hua-Qing Zhang, Jia-Qi Wang, Bo Zhang, Cai-Liang Shen

**Affiliations:** Department of Orthopedics, The First Affiliated Hospital of Anhui Medical University, Hefei, Anhui, China

**Keywords:** ferroptosis, intervertebral disc degeneration, iron, lipid peroxidation, therapeutic implication

## Abstract

Ferroptosis, an iron-dependent form of programmed cell death marked by phospholipid peroxidation, is regulated by complex cellular metabolic pathways including lipid metabolism, iron balance, redox homeostasis, and mitochondrial activity. Initial research regarding the mechanism of ferroptosis mainly focused on the solute carrier family 7 member 11/glutathione/glutathione peroxidase 4 (GPX4) signal pathway. Recently, novel mechanisms of ferroptosis, independent of GPX4, have been discovered. Numerous pathologies associated with extensive lipid peroxidation, such as drug-resistant cancers, ischemic organ injuries, and neurodegenerative diseases, are driven by ferroptosis. Ferroptosis is a new therapeutic target for the intervention of IVDD. The role of ferroptosis in the modulation of intervertebral disc degeneration (IVDD) is a significant topic of interest. This is a novel research topic, and research on the mechanisms of IVDD and ferroptosis is ongoing. Herein, we aim to review and discuss the literature to explore the mechanisms of ferroptosis, the relationship between IVDD and ferroptosis, and the regulatory networks in the cells of the nucleus pulposus, annulus fibrosus, and cartilage endplate to provide references for future basic research and clinical translation for IVDD treatment.

## Introduction

Low back pain (LBP) is a common musculoskeletal disease in the world, and its prevention and treatment are the major challenges in public health programs, which contribute to severe socioeconomic and health burdens (1). Intervertebral disc (IVD) degeneration (IVDD) has been considered as the leading cause of LBP, thereby resulting in a series of structural changes, such as the decrease of intervertebral height, breakage of the existing nucleus pulposus (NP), fissure of annulus fibrosus (AF), calcification of cartilage endplate (CEP), and imbalance of extracellular matrix (ECM) metabolism (2). In recent years, many new ways of programmed cell death have been reported in studies on IVDD. In contrast to apoptosis, necroptosis, pyroptosis, autophagy, and other types of death procedures, ferroptosis is characterized by the iron-mediated accumulation of lipid peroxides, morphologically manifested as mitochondrial shrinkage, reduction of mitochondrial cristae, and rupture of the mitochondrial outer membrane, and it has been regarded as a new target for the treatment of IVDD (3).

The overload of cellular iron content, particularly ferrous iron, can induce lipid peroxidation of fatty acids (4). The abnormal mitochondrial oxidative phosphorylation pathway results from iron overload, which produces a large amount of reactive oxygen species (ROS) and ATP. When the ROS content exceeds the scavenging level of the antioxidant system, polyunsaturated fatty acids (PUFAs) on the cell membranes and organelle membranes are oxidized to form lipid peroxides, which directly or indirectly destroy cell structure and function, thereby resulting in cell damage or death. Initial research on the mechanism of ferroptosis primarily focuses on the solute carrier family 7 member 11 (SLC7A11)–glutathione (GSH)–glutathione peroxidase 4 (GPX4) signaling pathway. Recently, novel mechanisms of ferroptosis independent of GPX4 have been discovered, which are closely related to lipid metabolism, iron balance, and redox reactions.

Although ferroptosis has been extensively investigated in various physiological and pathological processes, such as tumors, injuries, viral infection, immune response, and metabolic disorders since the item was coined by Dixon et al. (5) in 2012, research regarding the relationship between ferroptosis and IVDD started relatively lately (6–9). To date, a growing number of studies have investigated the relationship between ferroptosis and IVDD. Herein, we aimed to review recent literature to explore the underlying mechanism of ferroptosis and its role in IVDD and to investigate new therapeutic targets for the treatment of IVDD.

## Iron metabolism

### Systemic iron homeostasis

**Figure 1:**
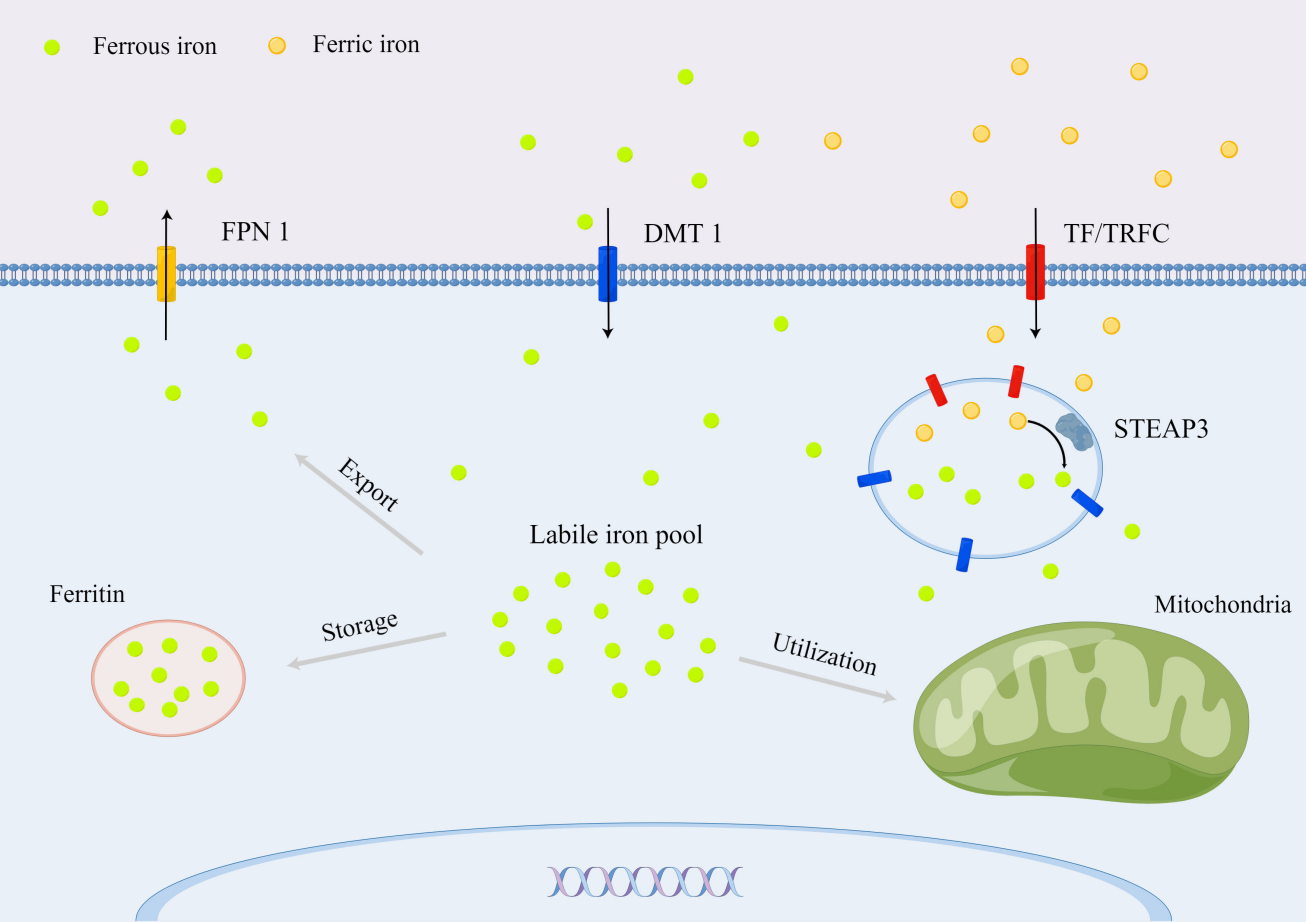
Cellular iron metabolism in mammals.

Iron homeostasis is essential for various metabolic processes in mammalian organ systems **(** **Figure 1**). Iron absorption mainly has two sources (heme iron primarily from animal products, including beef, fish, chicken, and liver, and non-heme iron primarily from fruit, vegetables, eggs, and grains) in the intestine, depending on different receptors (10). The heme iron is transported through the intestinal epithelium by heme carrier protein 1 (11). For the non-heme iron, ferric iron is reduced to be ferrous iron by cytochrome b reductase 1, which is then transported by divalent metal transporter 1 (DMT1), a carrier protein, into the enterocytes (12). Ferrous iron is exported through the iron exporter, ferroportin 1 (FPN1) (12, 13). The ferrous iron is oxidized from +2 to +3 state by hephaestin, subsequently loading ferric iron onto transferrin (TF) for systematic transport in the bloodstream (14). Moreover, the systematic iron homeostasis is complemented by serum ferritin and non-TF bound iron and regulated by the hepcidin–FPN1–regulatory axis (15).

For intracellular iron homeostasis, ferric iron binding to the TF in the serum can be taken up by a transferrin receptor (TFRC) on the cell membrane (16). The ferric iron is released from the TF in the endosome because of the rapid drop of pH and then reduced by six transmembrane epithelial antigens of prostate 3 (STEAP3) to ferrous iron, which is subsequently transported into the cytoplasm through the solute carrier family 11 member 2 (SLC11A2)/DMT1 (17). The transported ferrous iron stored in ferritin or labile iron pool for further utilization is essential for metabolic and biochemical processes, such as the regulation of the iron-requiring enzymatic activity, iron–sulfur protein production, and oxygen transport (18). Excess iron can be extruded into the extracellular space *via* the iron-efflux protein metal transporter protein-1/FPN1/iron-regulated transporter-1, which is the product of the solute carrier family 40 member 1 (SLC40A1) gene (19). Moreover, the intracellular iron homeostasis is regulated by iron-responsive element binding protein 2, heme oxygenase 1 (HO-1), and iron regulatory proteins (20, 21).

### Iron overload in the blood circulation

Hematological disorders, such as hereditary hemochromatosis associated with gene mutations of HFE, hepcidin hormone, and TFRC, can contribute to a high serum ferritin level (22, 23). In addition, chronic renal failure receiving repeated hemodialysis and other chronic diseases receiving repeated blood transfusions, including myelodysplastic syndrome and thalassemias, can saturate the iron-binding capacity of TF in the cytoplasm, leading to chronic iron overload (24, 25).

### Intracellular iron overload

Restrictive export and excessive import of iron result in intracellular iron overload. Genetic defects in SLC40A1 and STEAP3 mutations restrict iron export, but they have no effect on iron import (26, 27). Genetic mutations in SLC11A2 accelerate iron import, leading to intracellular iron overload (28).

### Iron overload in IVD

Iron accumulation in IVD is commonly observed in aging patients suffering from diseases because of the lack of effective mechanisms to exert excess iron, including hereditary hemochromatosis and thalassemia (29, 30). Meanwhile, iron overload in IVD may result from neovascularization within the disc, which exposes tissues to high levels of heme, a major source of intracellular iron (31, 32). Neovascularization was initially reported in herniated NP using histological staining in 1993, and Shan et al. (31) found that the immature vessels during neovascularization in herniated IVD lead to the extravasation of red blood cells and the deposition of iron in this tissue.

## Signaling pathways of ferroptosis

### SLC7A11/GSH/GPX4 signaling pathway

**Figure 2:**
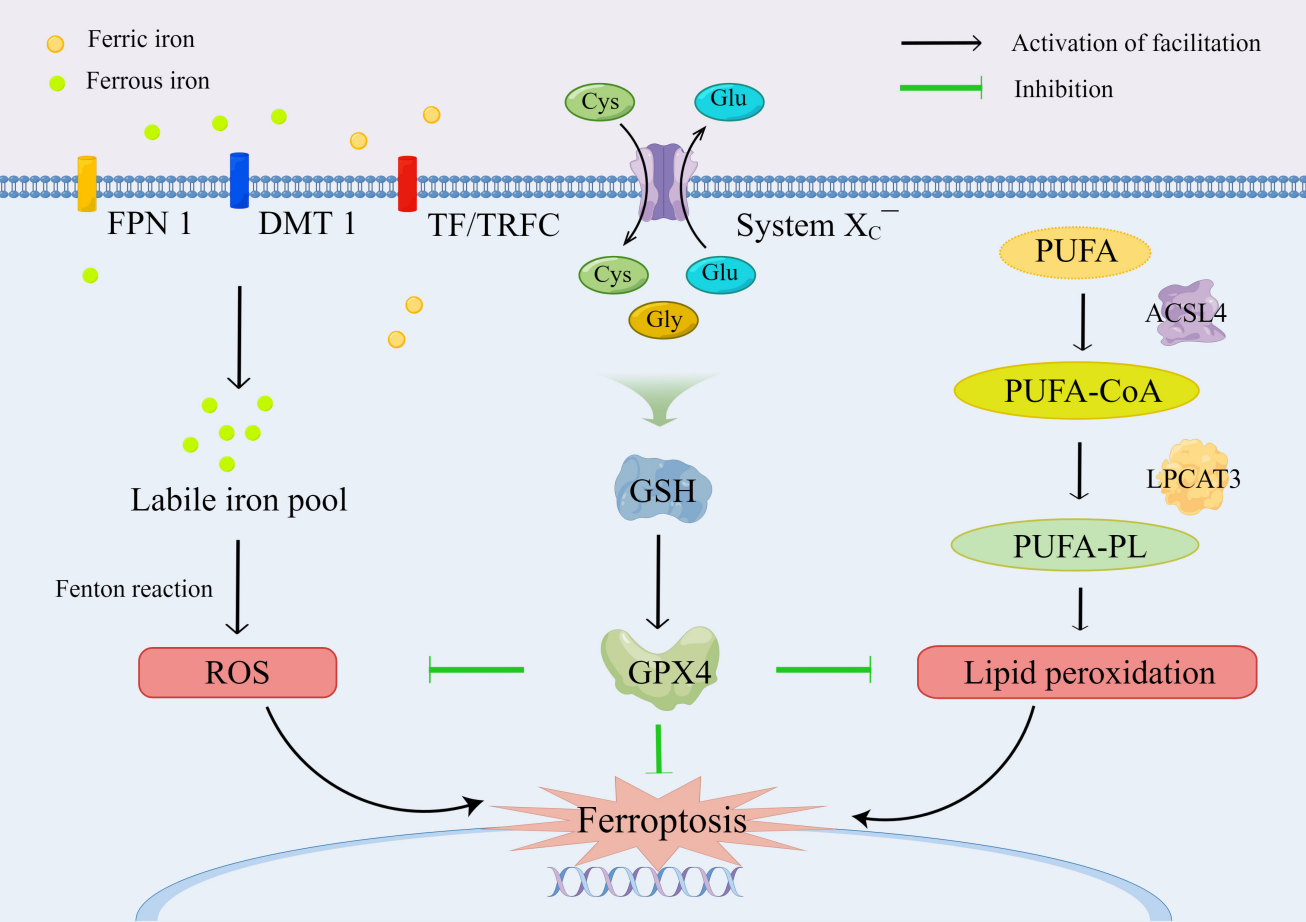
The molecular mechanism and regulation of ferroptosis.

System X_C_¯, consisting of SLC7A11 and solute carrier family 3 member 2 (SLC3A2), is a Na^+^-dependent amino acid antiporter that is widely distributed in the plasma membrane and is responsible for the import of extracellular cystine and the export of intracellular glycine (33). Intracellular cystine is immediately reduced back to cysteine by depleting NADPH, which is a rate-limiting precursor amino acid for the synthesis of GSH, a tripeptide consisting of cysteine, glutamate, and glycine (34). GSH plays an important role in anti-oxidative stress, reduction of lipid peroxidation, and protection of tissue cells, which is a necessary cofactor of GPX4 for normal function (35). Compared with other members of the GPXs family, GPX4 can directly convert phospholipid hydroperoxides (PLOOHs), a form of lipid-based ROS, on cell membranes to nontoxic lipid alcohols (PLOHs) with sufficient cellular GSH, whereas the depletion of GSH results in the inactivation of GPX4 (36, 37) (**Figure 2**).

GPX4 is the major neutralizing enzyme for PLOOHs, which protects the structure and function of cell membranes, and it has been regarded as a specific marker of ferroptosis, which plays an essential role in limiting lipid peroxidation (36, 38). Selenocysteine is the key group for the catalytic function of GPX4. PLOOH is reduced to PLOH, whereas the selenocysteine is oxidized to selenic acid intermediate (GPX4-SeOH). Subsequently, the selenium–glutathione adduct is produced after the reaction between GPX4-SeOH and GSH. Then, the selenium–glutathione adduct is converted back to selenocysteine by reacting with the equivalent of GSH. Similarly, the oxidized glutathione (GSSG) is produced from GSH, which is then reduced to GSH by glutathione reductase for recycling and utilization (35). Apart from ferroptosis, GPX4 plays a role in pyroptosis (39), apoptosis (40), necroptosis (41), and autophagy (42, 43), indicating that the regulation of PLOOHs may be a hallmark in the signaling pathway for the induction of regulated cell death.

Recently, the regulation of ferroptosis through the SLC7A11/GSH/GPX4 signaling pathway has been explored for the intervention of IVDD. Zhang et al. (44) demonstrated that the promotion of methylase expression upregulated GPX4 methylation in patients with hyperhomocysteinemia (HHcy), thereby inducing ferroptosis in NP cells (NPCs). In addition, the level of GPX4 protein was reduced after treatment with heme by simulating neovascularization in a heme-induced ferroptosis model (31). Moreover, ferroptosis in cartilage cells was modulated *via* the IL-6/miR-10a-5p/IL-6R axis in the inflammatory microenvironment (45), and the IL-6/STAT3/GPX4 signaling pathway might be implicated in this procedure (46).

### Signaling pathways independent of GPX4

**Figure 3:**
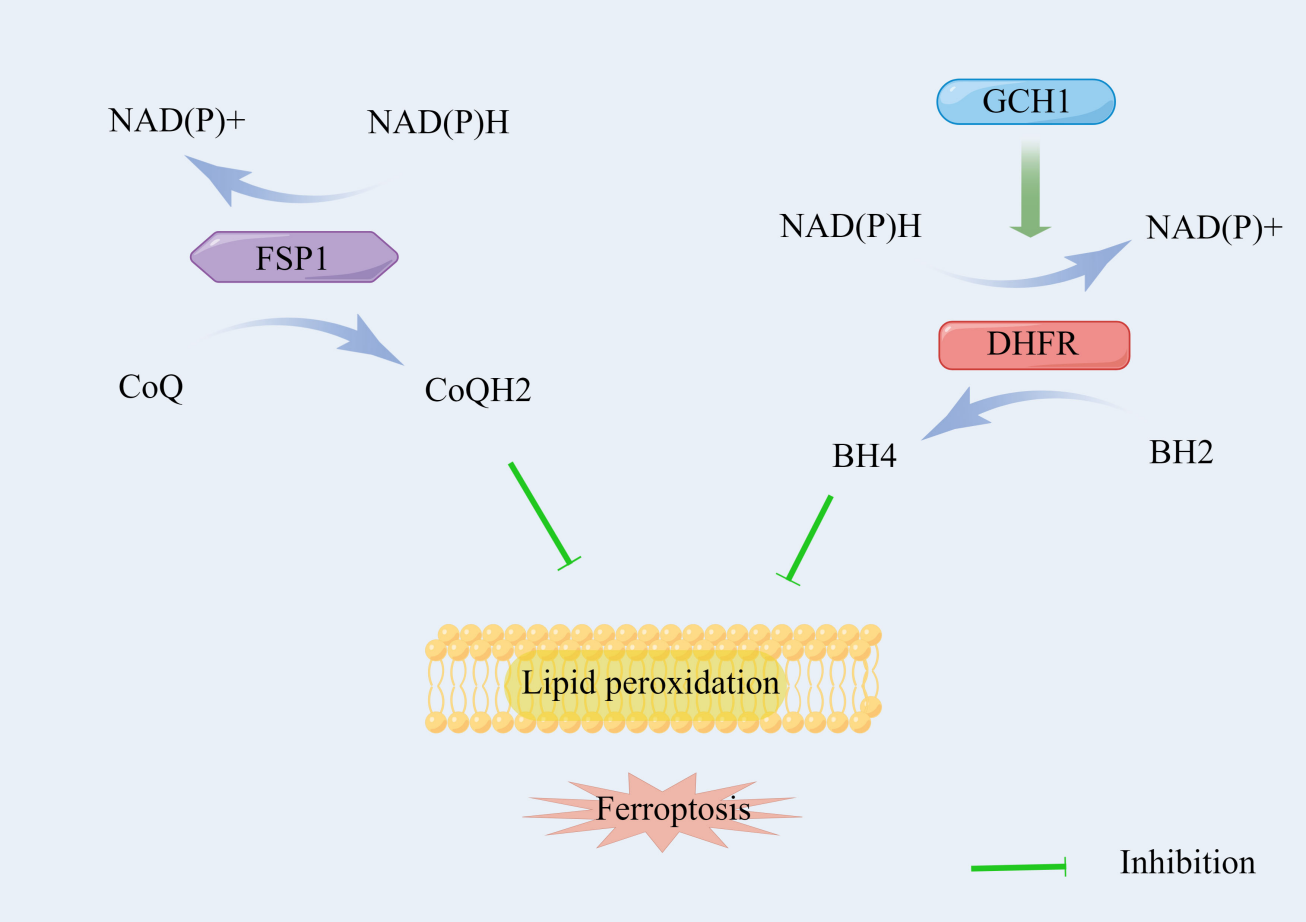
The signaling pathways of ferroptosis independent of SLC7A11/GSH/GPX4 axis.

GPX4 has been regarded as the primary enzyme that prevents ferroptosis through the conversion of lipid hydroperoxides into non-toxic lipid alcohols (36). However, the sensitivity of GPX4 inhibitors differs in cancer cell lines, indicating that additional independent pathways govern the regulation of ferroptosis (47). Therefore, current mechanisms of intracellular defense against ferroptosis can be divided into the SLC7A11-GSH-GPX4 signaling pathway and other signaling pathways independent of GPX4 **(** **Figure 3** **).**

Bersuker et al. (48) and Doll et al. (49) identified that ferroptosis suppressor protein 1 (FSP1), also known as apoptosis-inducing factor mitochondrial 2, acts parallel to the GSH-dependent GPX4 pathway with regard to the inhibition of phospholipid (PL) peroxidation and ferroptosis. FSP1 on the membrane reduces coenzyme Q (CoQ) by using NAD(P)H to ubiquinol (CoQH2), which serves as a lipophilic radical-trapping antioxidant (RTA), suppressing the propagation of lipid peroxides (50, 51). The loss of FPS1 improves PL peroxidation with the normal function of GPX4, indicating an independent mechanism of the FSP1/CoQH2/NAD(P)H pathway during ferroptosis.

The GTP cyclohydrolase-1 (GCH1)/6(R)-L-erythro5,6,7,8-tetrahydrobiopterin (BH4)/dihydrofolate reductase (DHFR) axis is another unique protective mechanism for ferroptosis, which is independent of the GSH-GPX4 system. Kraft et al. (52) identified GCH1 as a potent antagonist of ferroptosis using a whole-genome activation screen. The endogenous antioxidant BH4 on the membrane generated by the enzyme GCH1 also serves as a lipophilic RTA to selectively neutralize PUFA-PL-OOH, which alleviates sensitivity to ferroptosis. Moreover, BH4 also participates in the synthesis of CoQ to leverage oxidative damage under oxidative stress (52). Furthermore, DHFR serves as an essential regulator of ferroptosis by regenerating BH4 from dihydrobiopterin (BH2). The genetic or pharmacological loss of DHFR’s function can also induce ferroptosis (53).

## Metabolism and ferroptosis

### Lipid metabolism and ferroptosis

Lipid peroxidation is an important process in ferroptosis. PUFA-containing PLs on the cell membrane are easily oxidized because of the highly active hydrogen atoms in the methylene bridge, destroying the structure and stability of the lipid bilayer, and disintegration of the cell membrane. Free PUFAs, such as adrenal acid and arachidonic acid, are catalyzed by acyl-CoA synthetase long-chain family member 4 (ACSL4) to generate PUFA-CoA, which is then transported to the cell membrane through lysophosphatidylcholine acyltransferase 3 (LPCAT3) by inserting acyl groups into lysophospholipids and synthesizing PUFA-PLs with PLs (54–56). PUFA oxidation mainly has two forms. First, PUFA can be oxidized through an enzymatic reaction. PUFA-PL is catalyzed by arachidonate lipoxygenase (ALOX) into PUFA-PL-OOH (57). Zou et al. (58) found that cytochrome P450 oxidoreductase (POR) promotes lipid peroxidation during ferroptosis in an ALOX-independent manner using systematic lipidomic profiling and suggested that POR is an essential mediator of ferroptosis. In addition, PUFAs are oxidized by other oxygenases, including NADPH oxidases (NOXs) and prostaglandin-endoperoxide synthase 2 (PTGS2/COX) (59, 60). Despite being upregulated during ferroptosis, PTGS2 might not be involved in the production of lipid peroxidation. Whether PTGS2 affects the procedure of ferroptosis still needs further investigation (61). Second, PUFA oxidation occurs through Fenton reaction in a non-enzymatic way. Ferric iron, hydroxyl radicals (HO ·), and OH- are generated during the reaction between ferrous iron and hydrogen peroxide (H_2_O_2_). Thus, free radical ions further cause oxidative damage to membrane lipids, particularly PUFAs. The inhibition of key molecules, such as ACSL4, LPCAT3, ALOX, POR, and NOXs, is of great significance to the reduction of lipid peroxidation, thereby counteracting ferroptosis.

### Iron metabolism and ferroptosis

The main mechanism of the biological toxicity of iron ions is the classical Fenton reaction, where ferrous iron reacts with hydrogen peroxide (H_2_O_2_). Among the products of the Fenton reaction, the hydroxyl radicals are largely destroyed, which can not only cause oxidative damage to cells by unspecifically attacking biomolecules, but also promote the peroxidation of lipid components to generate various oxidation products, the main products of which are lipid hydroperoxides (LOOHs) (57). LOOHs can be converted to oxygen radical intermediates, including lipid peroxyl radical (LOO ·) or alkoxyl (LO ·) (62). Given the high proportion of PUFAs on cell and plasma membranes, the oxygen radical intermediates cause cascade reactions, which further aggravate the destruction of the membranes, contributing to the disturbance of cellular homeostasis and activation of serious biochemical reactions. Furthermore, many different aldehydes that can be formed as secondary products, including 4-hydroxynonenal, malondialdehyde, hexanal, and propanal, can continuously react with PUFAs, destroy cells, and eventually lead to irreversible disruption of the structure and function of the cell membranes (63–65). Finally, the free radical ions and hydroxyl radicals generated from intracellular free ferrous ions through the Fenton reaction oxidize PUFA on the cell membrane and damage the protein in the cytoplasm and DNA in the nucleus (66). Moreover, iron ions are considered as the key components of various metabolic enzymes, including ALOX and POR. Therefore, homeostasis of iron ions is important for the normal functioning of organisms and cells.

The regulation of iron homeostasis can affect the sensitivity of cells to ferroptosis with regard to the uptake, storage, and efflux of iron. The knockdown of TF can suppress lapatinib-induced ferroptosis in SKBR3 cancer cell line, and the loss of TFRC can also decrease cystine starvation- or erastin-induced ferroptosis (67–69). Ferritin, composed of H and L subtypes, is the main iron storage protein primarily located in the cytoplasm, which stores around 70%–80% newly imported iron (35). The H subtype, ferritin heavy chain 1 (FTH1), can oxidize ferrous iron to ferric iron and combine with it to reduce free ferrous iron and subsequent Fenton reaction. SLC40A1, the only known iron exporter in mammalian cells, can influence ferroptosis by mediating iron output. Studies have shown that ferroptosis is promoted by the knockdown of SLC40A1, whereas this procedure is ameliorated by the overexpression of SLC40A1 (68, 70). In response to different types of ferroptosis inducers, the level of intracellular ferric ions will increase, and various protein transporters related to iron metabolism, such as TF, TFRC, ferritin, and SLC40A1, will be rearranged under the ferroptosis program.

In the oxidative stress microenvironment of IVDD simulated by the tert-butyl hydroperoxide (TBHP), Lu et al. (71) indicated that the intercellular iron overload resulted from FPN dysregulation that was regulated by metal-regulatory transcription factor 1 (MTF1), and the TBHP-indued ferroptosis was aggravated through the JNK/MTF1/FPN signal pathway. In addition, the levels of intercellular iron in AF cells (AFCs) and NPCs were increased by nuclear receptor coactivator 4 (NCOA4)-mediated ferritin selective autophagy during TBHP-indued ferroptosis (72).

### Animo acid metabolism and ferroptosis

Amino acid metabolism is an important part of the metabolic loop of organisms, and imbalances in intracellular and extracellular cysteine, cystine, glutamate, and GSH can induce ferroptosis. Intracellular cysteine is primarily used to synthesize antioxidant enzymes, including GSH and thioredoxin. When cysteine is deficient, cystathionine-β-synthase (CBS) and cystathionine gamma-lyase are activated under oxidative stress conditions, and cysteine is biosynthesized from methionine through the transsulfuration pathway, thereby reducing oxidative stress–induced ferroptosis (73). Liu et al. (74) demonstrated that the overexpression of CBS can confer ferroptosis resistance in ovarian cancer cells, and CBS has been identified as a new negative regulator of ferroptosis. By contrast, cysteinyl-tRNA synthetase (CARS) positively regulates ferroptosis by limiting the transsulfuration pathway. Hayano et al. (75) found that the loss of CARS contributed to the accumulation of cystathionine, induction of the transsulfuration pathway, and upregulation of genes associated with serine biosynthesis and transsulfuration.

The glutaminolysis pathway, in which glutamine is catabolized to glutamate, has also been implicated in the regulation of ferroptosis. Gao et al. (67) indicated that α-ketoglutarate converted from glutamine can cause cysteine deprivation and promote ferroptosis, and the limitation of glutaminolysis can reduce the heart triggered by ischemia–reperfusion injury through the inhibition of ferroptosis. Moreover, glutaminolysis is catalyzed by cytosolic glutaminase (GLS1) and mitochondrial glutaminase (GLS2); however, GLS2, instead of GLS1, is required for ferroptosis (67, 76, 77). Mitochondria play a crucial role in cysteine deprivation-induced ferroptosis, instead of GPX4 inhibition-induced ferroptosis, which is mediated by the potential hyperpolarization, mitochondrial tricarboxylic acid cycle, and electron transport chain of the mitochondrial membrane (78).

### Glucose metabolism and ferroptosis

Glucose is the principal nutrient for biosynthesis and the main source of acetyl-CoA for the synthesis of fatty acids. Under nutritional deficiency, energy stress is induced by the depletion of intracellular ATP and subsequent improvement of intracellular AMP levels. In short-term and slight energy stress, the AMP-activated protein kinase (AMPK), a sensor of cellular energy status, participates in the adaptive response by promoting ATP-generating catabolism and maintaining cell survival (79). Lee et al. (80) found that the energy stress caused by glucose starvation can partly reduce ferroptosis by AMPK, and the activation of AMPK inhibits ferroptosis by the phosphorylation of acetyl-CoA carboxylase and restrains PUFA biosynthesis. In type 2 diabetic osteoporosis, high glucose levels can induce ferroptosis *via* increased ROS/lipid peroxidation/GSH depletion (81).

## Transcriptional regulation of ferroptosis

The transcription factors regulate ferroptosis-related target genes that serve as promoters or blockers, thereby affecting the sensitivity of ferroptosis through multiple roles in transcription-dependent or transcription-independent mechanisms (82). Many transcription factors, such as tumor protein 53, nuclear factor-erythroid 2 like 2 (NRF2), Yes 1-associated transcriptional regulator, MTF1, activating transcription factor 3 (ATF3), transcription factor AP-2 gamma, specificity protein 1, hypoxia-inducible factor 1 alpha, and egl-9 family hypoxia-inducible factor 2, have been found to be involved in a ferroptotic network, which has been a new potential treatment target (83–89).

## Epigenetic regulation of ferroptosis

DNA methylation and histone modification can regulate ferroptosis. Helicase can inhibit ferroptosis by DNA methylation through the induction of sterol-CoA desaturase 1 and fatty acid desaturase 2, epigenetic silencing of cytosolic long non-coding RNAs (IncRNA) LINC00472, and promotion of nuclear lncRNA 00336 (35, 90–92). Histone 2A ubiquitination (H2Aub) and histone 2 B ubiquitination (H2Bub) can induce SLC7A11 expression by histone modification to reduce sensitivity to ferroptosis. Bromodomain containing 4 (BRD4) epigenetically prevents ferroptosis by recognizing acetylated lysine residues on histones, and demethylase 3 B (KDM3B), a histone H3 lysine 9 demethylase, can activate the expression of SLC7A11 to reduce erastin-induced ferroptosis (93, 94).

## Inducers and inhibitors of ferroptosis

**Figure 4:**
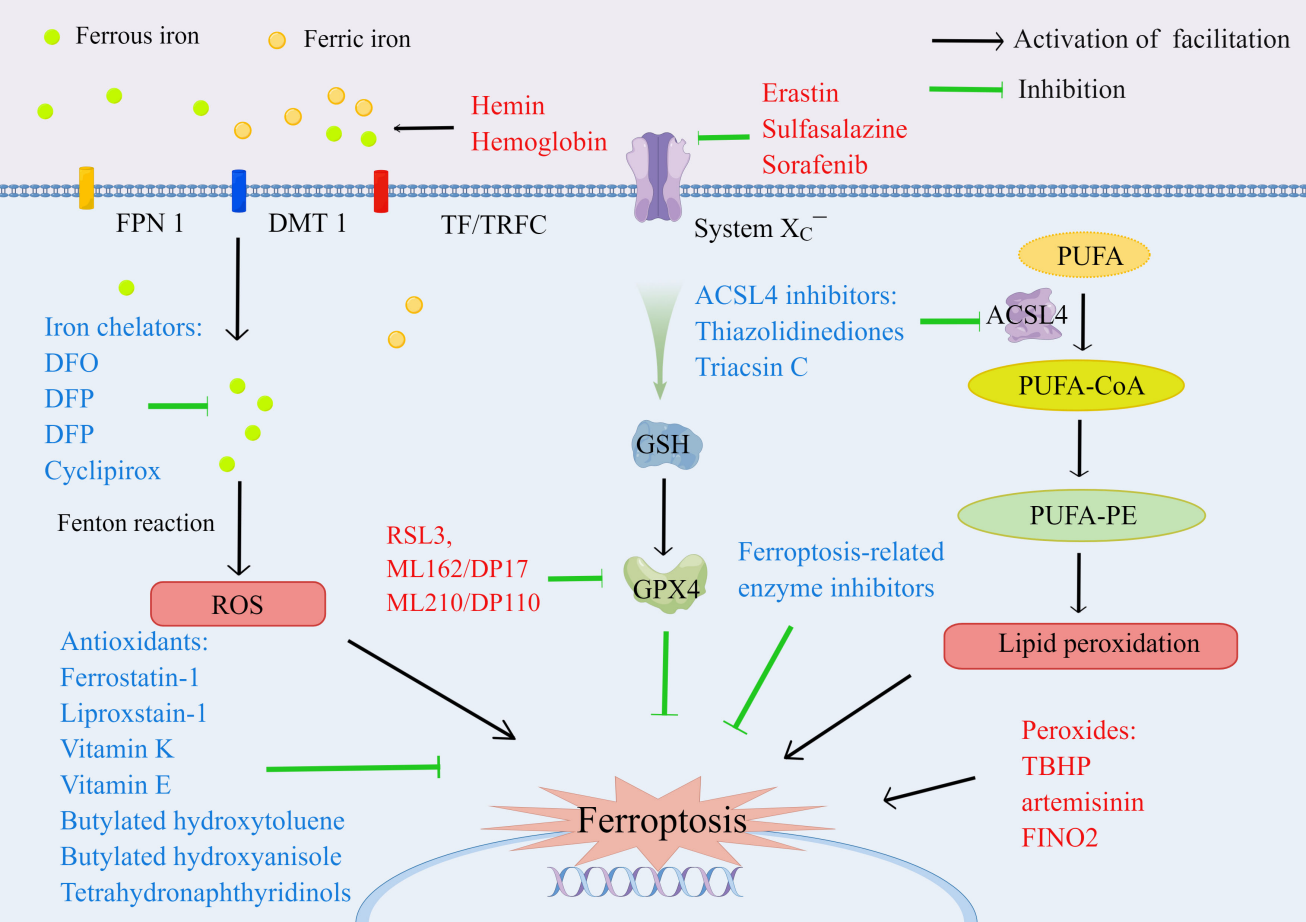
The inducers (marked red) and inhibitors (marked blue) of ferroptosis.

The ferroptosis inducer (FINs) can be divided into four categories: The first category is the inhibiting activity of SCL7A11. The FINs inhibit the function of System X_C_¯ to reduce the uptake of cystine and the synthesis of GHS, including erastin, sulfasalazine, and sorafenib (5, 36). The second category refers to the FINs inhibiting GPX4. The FINs, including RSL3, ML162/DP17, and ML210/DP110, covalently react with selenocysteine to inhibit the activity of GPX4 (36, 95). The third category refers to organic peroxides that cause oxidative damage, including TBHP, artemisinin, and FINO2 (96, 97). The fourth category refers to the FINs resulting in iron overload, including exogenous hemin and hemoglobin (31, 98, 99). The inhibitors of ferroptosis include antioxidants (e.g., butylated hydroxytoluene, butylated hydroxyanisole, tetrahydronaphthyridinols, ferrostatin-1, liproxstain-1, vitamin E, and vitamin K), iron chelators (e.g., deferoxamine [DFO], deferasirox [DFP], deferiprone [DFX], and ciclopirox), ferroptosis-related enzyme inhibitors (e.g., ALOX inhibitors, including baicalein, zileuton, and cinnamyl-3,4-dihydroxya-cyanocinnamate; ACSL4 inhibitors, including thiazolidinediones and triacsin C; and NOX inhibitors, including diphenylene iodonium and 2-acetylphenothiazine), and protein degradation inhibitors (5, 35, 100–103) **(** **Figure 4** **).**

## Ferroptosis and IVDD

The IVD is a special structure without blood vessels in an ischemic and hypoxic microenvironment under normal physiological conditions, the steady balance of which is the basis for the maintenance of normal function. IVDD is a chronic process that commonly causes LBP; however, the specific cause of IVDD remains unclear. Published studies have demonstrated that IVDD is a complex process with multifactorial interactions, which is primarily characterized by ECM destruction and cell phenotype changes, as well as apoptosis, autophagy, pyroptosis, necroptosis, and ferroptosis of IVD (31, 104–107). The term ferroptosis was first coined in 2012. Since then the molecular mechanism and regulatory network of ferroptosis have been exponentially investigated in many degenerative diseases, such as Parkinson’s disease, Alzheimer’s disease, kidney degeneration, atherosclerosis, osteoporosis, and osteoarthritis (OA) (108–113). Although studies on ferroptosis in IVDD have been conducted in recent years, increasing evidence has shown that ferroptosis is associated with IVDD and is involved in degenerative processes of NP, AF, CEP, and ECM (**Table 1**).

**Table 1:** Mechanism and intervention methods of ferroptosis in IVDD.

Study	Induction of ferroptosis	Mechanism	Effects on cells	Intervention method
Zhang et al. (44)	Hcy to simulate the pathological condition of HHcy	Promotion of methylase expression and the upregulation of the GPX4 methylation.	Inducing ferroptosis in NPCs	Folic acid reducing the ability of HHcy to promote IVDD
Bin et al. (45)	IL-6 to simulate inflammatory condition	IL-6/miR-10a-5p/IL-6R axis	Inducing ferroptosis in cartilage cells	Inhibiting miR-10a-5p and subsequently derepressing IL-6R signaling pathway
Lu et al. (71)	TBHP to simulate oxidative stress condition	FPN downregulation and intercellular iron overload	Inducing ferroptosis in NPCs.	Enhancing the nuclear translocation of MTF1 by suppressing the JNK pathway and ameliorating the progression of IVDD
Shan et al. (31)	Heme to simulate neovascularization condition	Increased heme catabolism, downregulation of GPX4, and intercellular iron overload, which might be mediated by the Notch pathway	Inducing ferroptosis in NPCs.	Inhibiting of the Notch signaling pathway
Yang et al. (72)	TBHP to simulate oxidative stress condition	NCOA4-mediated ferritinophagy and intercellular iron overload	Inducing ferroptosis in NPCs and AFCs.	Silencing NCOA4 to alleviate ferroptosis
Li et al. (114)	TBHP to simulate oxidative stress condition	Upregulation of ATF3 and ROS products	Inducing ferroptosis in NPCs.	Silencing ATF3 by miR-874-3p and alleviating IVDD
Wang et al. (115)	FAC to simulate iron overload condition	Mineralization of endplate chondrocytes and oxidative stress	Inducing ferroptosis in endplate chondrocytes.	DFO, NAC and ferrostatin-1 rescuing high dose iron-induced IVDD and cartilage endplate calcification
Yu et al. (116)	IL-1β to simulate inflammatory condition	Decreased NRF2 expression and upregulation of ROS products	Inducing ferroptosis in NPCs.	circ_0072464 shuttled by BMSC-secreted EVs inhibiting NPC ferroptosis by downregulation of miR-431 and upregulation of NRF2.

IVDD, Intervertebral disc degeneration; Hcy, homocysteine; HHcy, hyperhomocysteinemia; NPCs, nucleus pulposus cells; TBHP, tert-butyl hydroperoxide; FPN, ferroportin; MTF1, metal-regulatory transcription factor 1; GPX4, glutathione peroxidase 4; NCOA4, nuclear receptor coactivator 4; AFCs, annulus fibrosus cells; ATF3, activation transcription factor 3; ROS, reactive oxygen species; FAC, ferric ammonium citrate; DFO, deferoxamine; NAC, N-acetyl-cysteine; NRF2, nuclear factor-erythroid 2 like 2; BMSC, bone marrow mesenchymal stem cells; EV, extracellular vesicle.

### Ferroptosis in NP

The property of resident progenitor cells in NP is altered by IVDD (117). The healthy NP primarily consists of chondrocyte-like cells, whereas the degenerative NP is primarily composed of chondrocyte-like cells, inflammatory cells, and fibroblast-like cells, which shrank extensively and became yellowish and fibrous (118). Zhang et al. (119) performed single-cell RNA sequencing analysis of NPCs isolated from normal controls and from patients with IVDD. Gene Ontology and Kyoto Encyclopedia of Genes and Genomes analyses revealed that ferroptosis pathways were enriched in mild IVDD. The pathways identified by scRNA-Seq were validated using a rat model of IVDD, and the levels of iron (a sign of ferroptosis), FTL, and HO-1 (two important regulators of ferroptosis) were assessed. They found that NP in the degenerative group was associated with remarkably higher iron levels and lower levels of ferritin light chain and HO-1 than in the control group, indicating that ferroptosis played a role in the progression of IVDD. Shan et al. (31) found that the increased level of iron primarily resulted from the high level of heme caused by neovascularization in degenerative NP, thereby inducing cytotoxicity and ferroptosis and accelerating the progression of IVDD. This result is also supported by scRNA-Seq analysis reporting endothelial cells only in IVDD samples, and the proportion of endothelial cells increased with the severity of IVDD (119).

Ferroptosis is regulated by multiple pathways during IVDD. Lu et al. (71) detected a decreased expression of FPN and the occurrence of ferroptosis under oxidative stress conditions simulated using TBHP in human NPCs *in vitro* and *in vivo*. They found that the downregulation of FPN, but not TFRC and DMT1, primarily accounted for the intercellular iron overload and ferroptosis in TBHP-induced human NPCs, whereas the overexpression of FPN inhibited ferroptosis through the JNK/MTF1/FPN signaling pathway. Therefore, the decreased nuclear translocation of MTF1 under TBHP treatment contributes to the reduced expression of FPN and ferroptosis in human NPCs. Meanwhile, hinokitiol, a natural tropolone derivative, can increase the nuclear translocation of MTF1, restore FPN, and attenuate TBHP-induced ferroptosis by suppressing the JNK pathway in human NPCs, as well as in the NP tissue of IVDD. Moreover, ferritinophagy is involved in TBHP-induced ferroptosis of NPCs through NCOA4-mediated ferritin-selective autophagy in an autophagy-dependent manner (72). NCOA4, a selective cargo receptor mutually combining with ferritin, transports ferritin to the autophagosomes with the occurrence of oxidative stress in IVD cells, thereby releasing free iron to induce ferroptosis (72, 120, 121). A newly published clinical study also proved that serum ferritin was negatively correlated with the degree of IVDD, which can be used as a clinical predictor of IVDD severity (122). Furthermore, Shan et al. (31) showed that heme-induced ferroptosis of human NPCs by the inhibition of the GPX4 protein can be rescued by DFO treatment. In addition, heme-induced ferroptosis might be mediated by the Notch signaling pathway, with substantial changes in the mRNA and protein levels of Notch1, Notch2, Jag1, Jag2, Hes1, Hes2, and Hey1.

MicroRNAs (miRNAs) and short non-coding RNAs (ncRNAs) primarily downregulate the expression of target genes and modulate the related downstream pathways by directly binding to the 3′-untranslated regions of the target genes, thereby regulating ferroptosis in human NPCs and IVDD. Li et al. (114) established a rat model of IVDD with TBHP and found that the overexpression of ATF3 induced ROS production and ferroptosis by suppressing SLC7A11. In addition, bioinformatics analysis and molecular experiments demonstrated that ATF3 is a direct target of miR-874-3p, indicating that the upregulation of ATF3 partially results from the downregulation of miR-874-3p in IVDD. Extracellular vesicles (EVs) derived from most cell types have been increasingly considered as important mediators of cell-to-cell communication and biomarkers of diseases, and they are involved in pathophysiological processes of IVDD (123, 124). Exosome-transported circular RNAs (circRNAs) have also been confirmed to exert effects on the regulation of IVDD (125). CircRNAs serve as miRNA sponges, contributing to the downregulation of miRNA and upregulation of miRNA downstream targets (126). Yu et al. (116) found that the uptake of EVs extracted from mouse bone marrow mesenchymal stem cells (BMSCs) by NPCs alleviated IVDD. *In vitro* and *in vivo* experiments showed that circ_0072464 shuttled by BMSC-derived EVs reduced ferroptosis in NPCs through the inhibition of miR-431 and upregulation of miR-431-mediated NRF2, indicating a potential biotherapeutic target for the treatment of IVDD.

Ferroptosis of NPCs and IVDD is also regulated by DNA methylation. HHcy, characterized by increased total homocysteine in plasma and its close relation to DNA methylation, results from the high concentration of homocysteine (Hcy) in serum caused by the deficiency of folic acid or the excessive intake of methionine (127–129). Zhang et al. (44) demonstrated that Hcy aggravates oxidative stress and induces ferroptosis in NPCs through the promotion of methylase expression and upregulation of GPX4 methylation. They also confirmed that HHcy is an independent risk factor for IVDD and that HHcy accelerates IVDD *in vivo*, which can be rescued by folic acid and the methylase inhibitor 5-AZA.

### Ferroptosis in AF

AF, which is divided into the inner (proteoglycan and collagen II rich) and outer regions (collagen I rich), has strong resistance to traction and compression, preventing the NP from protruding outwards (130). The AFCs in the outer region tend to be fibroblast-like and parallel to collagen fibers, whereas the AFCs in the inner region can be more oval (131). Yang et al. (72) investigated the expression of ferroptosis marker proteins in the AFCs of a rat model exposed to TBHP at different concentrations. They found decreased expression of FTH and GPX4 and increased expression of PTGS2 and ACSL4 with an increase in TBHP concentration in AFCs, indicating the existence of oxidative stress–induced ferroptosis in rat APCs. Moreover, ferroptosis in AFCs is upregulated by NCOA4-mediated ferritinophagy in response to TBHP treatment, indicating new insights into the treatment of IVDD. The composition and structure of AF are unique and critical for the maintenance of disc anisotropy, elastic mechanical loading, and homeostasis. However, most published research focuses on NPCs, and studies on the effects of ferroptosis on AFCs are rare. Therefore, further studies on the relationship between AFCs must be conducted in the future.

### Ferroptosis in CEP

CEP interfaces the disc and vertebral body with a thin horizontal layer of semi-porous thickened cancellous bone and hyaline cartilage, serving as the predominant route for nutrition supply and waste product exchange within the IVD (131, 132). The degeneration of CEP has been regarded as the primary predictor of IVDD, which reduces tissue diffusivity and changes the biochemical microenvironment of IVD, leading to reduced glucose and oxygen concentrations, increased lactate levels, and decreased PH within the disc, thereby initiating IVDD (133–135). The overload of iron and ferroptosis plays a role in the degeneration and calcification of CEP. Wang et al. (115) explored the connection between iron overload and degeneration of CEP and found that oxidative stress mediated by iron overload induced endplate chondrocyte ferroptosis, which can be reversed by iron chelation, antioxidants, and ferroptosis inhibition, indicating that ferroptosis plays an important role in endplate chondrocyte degeneration. Bin et al. (45) demonstrated aberrant expression of interleukin (IL) 6 (IL-6) and its receptor in cartilage specimens obtained from patients with IVDD. Furthermore, they showed that cartilage cell ferroptosis is induced by IL-6 through oxidative stress and iron homeostasis. Furthermore, miR-10a-5p partially inhibited IL-6-induced ferroptosis by suppressing IL-6R expression, indicating that the IL-6/miR-10a-5p/IL-6R axis is a potential target for IVDD treatment.

Chondrocytes are the predominant cell type in ECP and articular cartilage. Thus, studies reporting the mechanisms of articular cartilage degeneration caused by ferroptosis in OA are inspiring and learnable for the intervention of IVDD. Jing et al. (136) indicated that iron overload in chondrocytes induced by pro-inflammatory cytokines contributes to oxidative stress and mitochondrial dysfunction through the upregulation of TRF1 and downregulation of FPN. In a study by Yao et al. (137), lipid ROS and ferroptosis in chondrocytes were induced by IL-1β and ferric ammonium citrate (FAC), but they were attenuated by Ferrostatin-1 that activated the NRF2 antioxidant system. The overexpression of NRF2 upregulated the level of GPX4 expression and ameliorated ferroptosis.

### Ferroptosis and ECM

Under normal circumstances, the components of the ECM in the IVD are continuously updated through anabolism and catabolism, and the cells in the IVD associated with ECM form a coordinated functional system (138). However, the imbalance of anabolic and catabolic activities might result in ECM degradation, which is a pathological characteristic of IVDD (139). ECM metabolism is modulated by iron overload and ferroptosis.

The ECM of NP primarily consists of collagen II, proteoglycan, and chondroitin sulfate (140). In NPCs, the levels of collagen II, proteoglycan, matrix metalloproteinases (MMPs, particularly MMP13), disintegrin, and metalloproteinase with thrombospondin motifs (ADAMTSs, particularly ADAMTS4 and ADAMTS5) can reflect the degree of ECM degradation during the progression of IVDD (141, 142). The overexpression of circ_0072464 can promote the levels of collagen II and proteoglycan and reduce the levels of MMP13 and ADAMTS5 by sponging miR-431, upregulating NRF2, and suppressing ferroptosis (116). Meanwhile, the overexpression of ATF3 in NPCs not only aggravates TBHP-induced ferroptosis, apoptosis, and ROS production by suppressing SLC7A11 and superoxide dismutase 2, but also enhances ECM degradation by reducing the levels of proteoglycan and collagen II (114).

In endplate chondrocytes, the iron overload induced by FAC treatment enhanced the expression of MMP3 and MMP13 and reduced the expression of collagen II, thereby accelerating the degeneration of CEP and ECM (115). The study conducted by Camacho et al. (143) also demonstrated that iron overload was involved in chondrocyte-mediated ECM degradation. Meanwhile, erastin, an inducer of ferroptosis, reduces the expression of collagen II and increases the expression of MMP13 in chondrocytes (137). Furthermore, the inflammatory factor IL-1β accelerated iron uptake in chondrocytes, which was then promoted after co-treatment with FAC, because IL-1β can promote the expression of TFRC and DMT1 but downregulate the expression of FPN1, thereby aggravating iron accumulation in chondrocytes (136). Finally, co-treatment with IL-1β and FAC upregulated the expression of ECM-degrading enzymes, including MMPs and ADAMTS5.

## Ferroptosis and IVDD treatment

Ferroptosis, characterized by iron-dependent lipid peroxidation and the accumulation of ROS within the IVD, is implicated in the pathogenesis of IVDD. Thus, ferroptosis opens a new therapeutic target for the intervention of IVDD with regard to the regulation of iron metabolism, chelation of iron, and antioxidants (113). Moreover, the key components in the signaling pathways are essential regulators of ferroptosis inhibition.

Classical iron chelators such as DFO, DFP, and DFX have been used for the clinical treatment of iron overload in thalassemia major (144). However, there is no clinical evidence of iron chelation therapy for the treatment of IVDD. Nevertheless, iron chelators have shown promising results in the inhibition of ferroptosis *in vivo* and *in vitro* in IVDD models. In addition, antioxidants, including Ferrostatin-1 and N-acetyl-cysteine (NAC), can exert protective effects against iron-induced abnormalities in IVDD. DFO and Fer‐1 reversed the decreased expression of FTH and GPX4 and the upward levels of autophagy and ferritinophagy induced by TBHP treatment in NPCs and AFCs (71, 72). In the tissue of CEP, the administration of DFO, NAC, and Ferrostatin-1 substantially inhibited iron overload-induced IVDD by alleviating endplate calcification and IVD collapse in a mouse model (115). Meanwhile, the FAC-induced ECM degradation and the decrease of mitochondrial membrane potential can be reversed by DFO or NAC (136). Furthermore, DFO partially reduced the inhibition of IL-6 on miR10a-5p in cartilage cell ferroptosis through the promotion of GPX4 and FPN1 and suppression of DMT1 expression (45). However, the long-term use of iron chelators may lead to iron deficiency in cells, which is detrimental to cellular metabolism. Thus, the safe application of iron chelators needs further investigation.

EVs, exosomes, and ncRNAs are involved in the regulation of gene expression related to ferroptosis, which has emerged as a potential therapeutic strategy for IVDD (116, 145, 146). The upregulation of miR-10a-5p inhibited IL-6R expression, thereby partially reducing IL-6-induced ferroptosis in chondrocytes (45). In addition, circ_0072464 shuttled by BMSC-secreted EVs suppresses ferroptosis in NPCs through the upregulation of miR-431-mediated NRF2 (116). At present, considerable research is needed to identify new ncRNAs and related mechanisms for the treatment of IVDD. Furthermore, the exact route of administration, safe dosing, and related dose toxicity of EVs, exosomes, and ncRNAs remain major problems in the application of regenerative medicine in clinical practice.

In addition, previous studies have demonstrated that hinokitiol ameliorates the activation of protein kinase B and mitogen-activated protein kinase to inhibit platelet activation and alleviate ferroptosis-related neurotoxicity through iron chelation and regulation of the NRF2 pathway (147, 148). Recently, Lu et al. (71) indicated that hinokitiol alleviates IVDD by upregulating MTF1, restoring FPN, and suppressing the JNK pathway, thereby attenuating TBHP-induced NPC ferroptosis. Furthermore, folic acid, a coenzyme in the methionine cycle, has been regarded as another ferroptosis-related therapeutic drug for IVDD caused by Hcy through the downregulation of GPX4 methylation and oxidative stress, thereby rescuing ferroptosis-induced NPC degeneration (44). In addition, other antioxidants and drugs, such as vitamin E, vitamin K, and curcumin, have exerted protective effects on ferroptosis (149–152). Moreover, the essential regulators of ferroptosis, such as ferritin, FPN1, NRF2, GPX4, GSH, HO-1, and TFRC, can be selected as the regulation targets for the treatment of IVDD. However, studies concerning the therapeutic effects of new bioactive compounds on ferroptosis-induced IVDD are rare and worthy of further studies.

The therapeutic efficacy of regulating iron homeostasis and ferroptosis to alleviate osteoporosis and OA was regarded as a potential option and reference for the treatment of IVDD. Icariin, the main active ingredient of Herba Epimedii, has antioxidant and antiosteoporosis functions by preventing iron overload-induced bone loss and regulating iron accumulation *in vitro* and *in vivo* (153). Icariin also attenuated IL-1β-induced degeneration of ECM and ROS in human OA cartilage *via* the activation of the Nrf2/ARE signaling pathway (154). Resveratrol, a member of the stilbene family of phenolic compounds, can reverse iron overload-induced bone loss by upregulating the levels of FOXO1 in osteoporotic mice (155). In addition, Tian et al. (156) demonstrated that NAC exerted protective effects against iron-mediated mitochondrial dysfunction and protected osteoblasts from iron overload-induced apoptosis. Moreover, melatonin (N-acetyl-5-methoxytryptamine), an effective endogenous antioxidant, can suppress high-glucose-induced ferroptosis by activating the Nrf2/HO-1 signaling pathway to improve bone microstructure in individuals with type 2 diabetes and osteoporosis (81). Furthermore, the upregulation of mitochondrial ferritin reduced osteoblastic ferroptosis under a high-glucose environment, whereas the deficiency of mitochondrial ferritin induced mitophagy *via* the ROS/PINK1/Parkin pathway in individuals with type 2 diabetes and osteoporosis (157). Therefore, mitochondrial ferritin might be another potential target for the treatment of type 2 diabetes and osteoporosis. Moreover, the endothelial cell‐secreted exosomes antagonized glucocorticoid‐induced osteoporosis *in vitro* and *in vivo via* the suppression of ferritinophagy‐dependent ferroptosis (158). Lu et al. (159) also indicated that EVs from endothelial progenitor cells suppressed the ferroptotic pathway of osteoblasts by restoring levels of GPX4 and System X_C_¯. Collectively, the pathophysiological progression of osteoporosis and OA is associated with iron metabolism disorder, ROS, and lipid peroxidation, which might provide potential therapeutic strategies for IVDD.

## Conclusions and perspectives

As a highly disabling disease, IVDD has attracted increasing attention worldwide. Growing evidence has shown that ferroptosis is involved in the pathophysiological processes of IVDD; thus, regulation of ferroptosis has become a new therapeutic target for IVDD. In this review, we summarized the pathogenesis and mechanisms of ferroptosis, the relationship between ferroptosis and IVDD, and the choice of IVDD treatment by inhibiting ferroptosis. Recent studies have demonstrated that ferroptosis is mainly regulated by SLC7A11/GSH/GPX4, FSP1/CoQH2/NAD(P)H, and GCH1/BH4/DHFR pathways. Ferroptosis is accompanied by metabolic imbalances of lipids, iron, amino acids, and glucose and is modulated by transcriptional and epigenetic regulation. The interaction and crosstalk between ferroptosis and IVD components in terms of NP, AF, CEP, and ECM provide remarkable insights into the prevention and treatment of IVDD. However, studies on ferroptosis in IVDD are still at a relatively early stage, and simulations of ferroptosis in IVDD are mainly induced by oxidative stress and inflammation. Other factors, such as hypoxia, acidic microenvironments, and compression, are needed to confirm the universality of ferroptosis in IVDD. Therefore, further research is required to investigate the specific mechanisms, molecular targets, and associated signaling pathways of ferroptosis to develop further understanding and effective options for intervention in IVDD.

## Author contributions

L-PZ, R-JZ and C-LS conceptualized the review. L-PZ and R-JZ drafted the manuscript. L-PZ, R-JZ, C-YJ, LK, Z-GZ, H-QZ, J-QW, BZ, and C-LS revised the manuscript. All authors have read and approved the final manuscript.

## Funding

This work was supported by the National Natural Science Foundation of China (No. 81772408).

## Acknowledgments

Figures were created by Figdraw (www.figdraw.com). We would like to thank Figdraw for its help in creating the figures.

## Conflict of interest

The authors declare that the research was conducted in the absence of any commercial or financial relationships that could be construed as a potential conflict of interest.

## Publisher’s note

All claims expressed in this article are solely those of the authors and do not necessarily represent those of their affiliated organizations, or those of the publisher, the editors and the reviewers. Any product that may be evaluated in this article, or claim that may be made by its manufacturer, is not guaranteed or endorsed by the publisher.
